# Supplementary material for: Altered microRNA Transcriptome in Cultured Human Liver Cells upon Infection with Ebola Virus
Source: Int J Mol Sci. 2021 Apr 6;22(7):3792. doi: 10.3390/ijms22073792 (PMC8038836; doi:10.3390/ijms22073792)
Supplement: Supplementary file 1 [file ijms-22-03792-s001.zip › Supplementary_File/C_ GO_Analysis_Results/16-30nt_go_Mayinga-96h-Huh7_vs_Control-96h-Huh7_up.mature_mirna_targets/MF_Pvalue_tree.pdf]

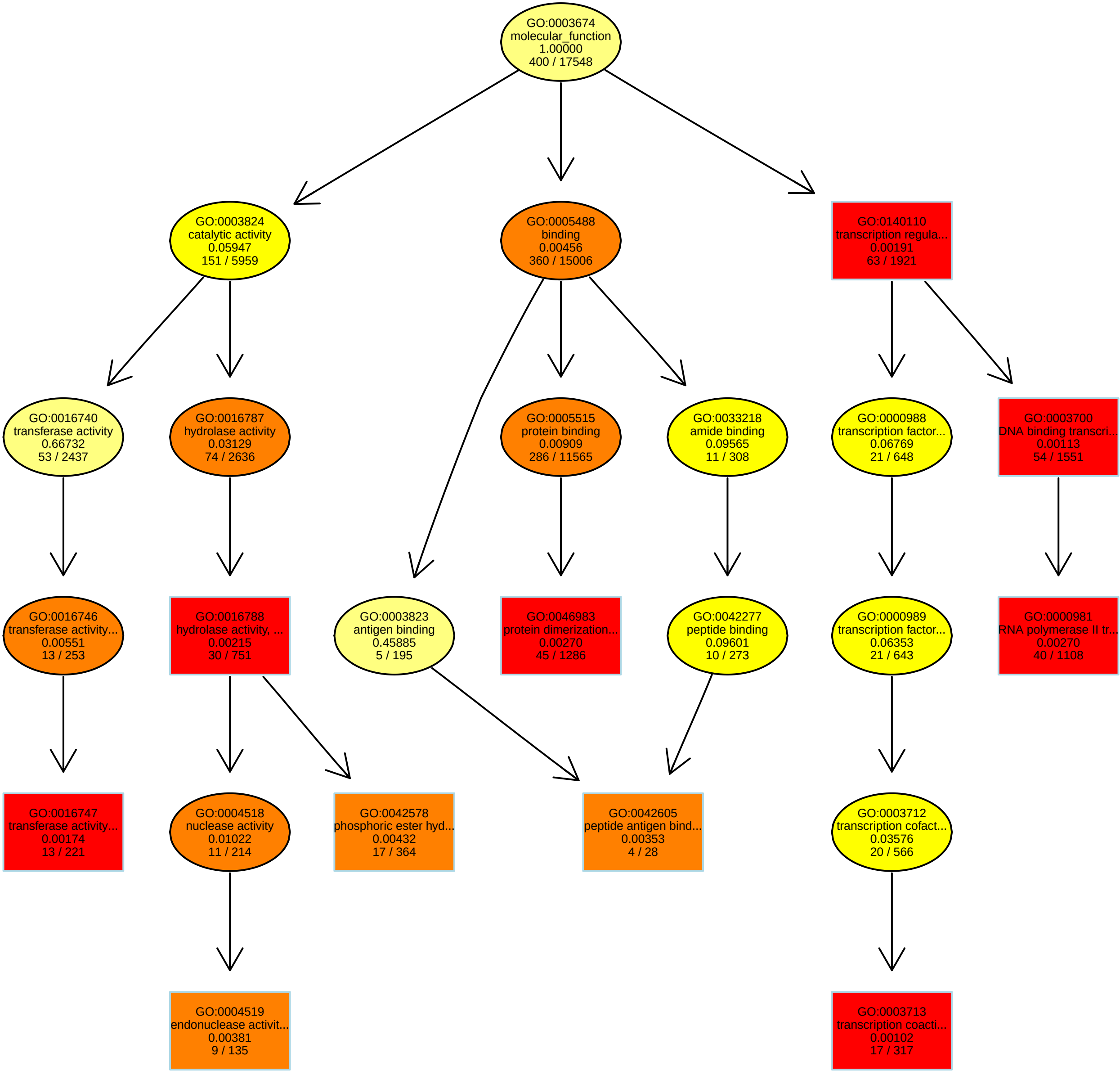
GO:0003674
molecular_function
1.00000
400 / 17548
GO:0003824
catalytic activity
0.05947
151 / 5959
GO:0005488
binding
0.00456
360 / 15006
GO:0140110
transcription regula...
0.00191
63 / 1921
GO:0016740
transferase activity
0.66732
53 / 2437
GO:0016787
hydrolase activity
0.03129
74 / 2636
GO:0005515
protein binding
0.00909
286 / 11565
GO:0033218
amide binding
0.09565
11 / 308
GO:0000988
transcription factor...
0.06769
21 / 648
GO:0003700
DNA binding transcri...
0.00113
54 / 1551
GO:0016746
transferase activity...
0.00551
13 / 253
GO:0016788
hydrolase activity, ...
0.00215
30 / 751
GO:0003823
antigen binding
0.45885
5 / 195
GO:0046983
protein dimerization...
0.00270
45 / 1286
GO:0042277
peptide binding
0.09601
10 / 273
GO:0000989
transcription factor...
0.06353
21 / 643
GO:0000981
RNA polymerase II tr...
0.00270
40 / 1108
GO:0016747
transferase activity...
0.00174
13 / 221
GO:0004518
nuclease activity
0.01022
11 / 214
GO:0042578
phosphoric ester hyd...
0.00432
17 / 364
GO:0042605
peptide antigen bind...
0.00353
4 / 28
GO:0003712
transcription cofact...
0.03576
20 / 566
GO:0004519
endonuclease activit...
0.00381
9 / 135
GO:0003713
transcription coacti...
0.00102
17 / 317
